# *Staphylococcus aureus* sequence type 71 is a chimera that emerged twice

**DOI:** 10.1186/s12864-026-12777-w

**Published:** 2026-04-18

**Authors:** Stefan Monecke, Sascha D. Braun, Celia Diezel, Elke Müller, Martin Reinicke, David C. Coleman, Hosny El-Adawy, Orla M. Keane, Amira A. Moawad, Renata Piccinini, Ralf Ehricht

**Affiliations:** 1 Leibniz Institute of Photonic Technology (IPHT), Leibniz Center for Photonics in Infection Research (LPI), Jena, Germany; 2 InfectoGnostics Research Campus, Jena, Germany; 3 Center for Translational Medicine (CETRAMED), Jena University Hospital, Friedrich Schiller University Jena, Jena, Germany; 4 Microbiology Research Unit, Division of Oral Biosciences, Dublin Dental University Hospital, University of Dublin, Trinity College Dublin, Dublin, Ireland; 5 Institute of Bacterial Infections and Zoonoses, Friedrich-Loeffler-Institut (Federal Research Institute for Animal Health), Jena, Germany; 6 Faculty of Veterinary Medicine, Kafrelsheikh University, Kafr El Sheikh, Egypt; 7 Animal and Bioscience Department, Teagasc, Grange, Dunsany, Co. Meath, Ireland; 8 Agriculture Research Center (ARC), Animal Health Research Institute, Giza, Egypt; 9 Department of Veterinary Medicine and Animal Science - DIVAS, University of Milan, Lodi, Italy; 10 Institute of Physical Chemistry, Friedrich-Schiller University, Jena, Germany

**Keywords:** *Staphylococcus aureus*, Horizontal gene transfer, Chimerism, Bacterial evolution, Next-generation sequencing

## Abstract

**Background:**

*Staphylococcus aureus* is a Gram-positive coccus that colonises or infects humans and a wide range of domestic and wild animals. Various lineages of *S. aureus* are adapted to ruminants, including cattle, where they commonly cause mastitis. This is of significant animal welfare and economic relevance. Clonal complex (CC) 97 is one of the *S. aureus* lineages found predominantly in bovines, but also in other animals and humans. Sequence Type (ST) 71 is a related bovine-associated lineage with part of its genome identical to CC97. Thus, it has been assumed that ST71 was a chimera consisting of a fragment of DNA from an unknown donor strain inserted into a CC97 backbone genome.

**Results:**

In this study, *S. aureus* ST3042 was identified as a putative donor of this fragment based on core genome multilocus sequence typing and alignment of core and genomic island markers. We also identified a second ST71 lineage from Sardinia that differs from previously known ST71 from Ireland, the United Kingdom and continental Europe, both in size and demarcations of the ST3042-derived insert. Sardinian ST71 sequences also differed from other ST71 in the orientation of a large part of the genome. As the inversion affected the ST3042-derived region, this event must have taken place later in time than the recombination of CC97 and ST3042.

**Conclusions:**

The observation that ST71 emerged twice adds to the body of evidence that recombination involving large segments of the genome is another, highly effective, mode of horizontal gene transfer in *S. aureus* and likely contributes to the extraordinary success of this pathogen.

**Supplementary Information:**

The online version contains supplementary material available at 10.1186/s12864-026-12777-w.

## Introduction

*Staphylococcus aureus* is a Gram-positive coccus that colonises 20–30% of any human population [1] but can also be found in domestic and wild [2, 3] animals. It is able to cause a wide range of infections, including skin and soft tissue infections (SSTIs), bone, joint and implant infections, pneumonia, septicaemia and various toxicoses. Various lineages of *S. aureus* are adapted to ruminants, including cattle, where they are a frequent etiological agent of mastitis causing a high economic burden with costs arising from veterinary treatments, animal culling, and the loss of milk production.

Mobile genetic elements play a significant role in the evolution of *S. aureus* as well as in host-adaption. Bacteriophages are extremely important in transmitting leukocidin genes [4–7], immune evasion genes [8, 9] and the exfoliative toxin gene A [10]. Staphylococcal Chromosome Cassette (SCC) elements are large genomic islands that encode recombinase genes and variable other payloads, commonly including the beta-lactam resistance genes *mecA/C* [11–15] or the fusidic acid resistance gene *fusC* [16, 17].

Mobile elements are not the only pathway to horizontal gene transfer utilised by *S. aureus.* Large-scale genomic replacements, or chimerism, might also contribute to a horizontal flow of genetic material. Huge parts of the genome can be transferred and integrated into *S. aureus* of other, frequently distantly related, lineages by a yet unknown mechanism(s). Multilocus-sequence typing (MLST) provides a convenient tool for detecting such cases. In MLST, seven housekeeping genes are defined, and their alleles are numbered [18]. If two strains or isolates have identical profiles, they belong to the same Sequence Type (ST). Non-identical, but related STs form Clonal Complexes (CCs). If two STs share, for instance, six alleles, but significantly differ in a seventh, this might indicate a recombination event. With increasing availability of whole-genome sequencing methods (and increasing computing power), schemes have been proposed expanding the MLST approach to the entirety of the non-mobile or conserved part of the bacterial genome, i.e., on more than 2,000 genes rather than just seven [19]. This approach is known as core genome MLST (cgMLST).

Previously reported examples of chimeric lineages include ST34 and ST42 (resulting from transfer of genomic DNA from CC10 into a CC30 genome [20, 21]), a CC1 x CC80 chimera from East Africa [22], several CC133-derived chimeras from waterfowl [23], ST239 (a pandemic methicillin-resistant *S. aureus* (MRSA) clone comprising CC30 genomic DNA integrated into a CC8 backbone genome [20]), a CC9 x CC398 chimera from European livestock [24], ST617 (a CC8 x CC45 chimera [25]), ST1048 and ST1774 (chimeric MRSA from Hong Kong [26]), ST2249 (a chimeric MRSA from Australia, derived from as many as three parental lineages: CC8, CC30 and CC45 [27]) and ST6610 (a CC8 x CC140 chimeric MRSA from East Africa [28]). ST71 is another chimeric lineage, first described from Irish livestock but also extant elsewhere in Europe [29–32]. It is clearly derived from the pandemic CC97 livestock lineage. However, it differs in several notable features, all located near the origin of replication (*oriC*). These include the absence of the histidine biosynthesis operon *his* and the biofilm-associated *ica* operon in ST71, the presence in ST71 of the collagen binding adhesin gene (*cna*) and of the enterotoxin-like gene ORF CM14 (corresponding to GenBank U10927.2, positions 32,627..33,406) or to locus tag SAB0026 in GenBank AJ938182.1; recently dubbed as *selz*: [33]). Canonical CC97 and ST71 furthermore differ in the allele of the MLST marker gene *arcC* (3 in ST97, 18 in ST71) and in alleles of other genes such as *lmrP.* They also differ in capsule type (5 in ST97, 8 in ST71). These differences are consistent with the replacement of a large fragment of more than 300,000 bp of the CC97 genome with a segment originating from a different strain [30]. In addition, a 30,000 bp fragment from this region that encompasses the *his* and *ica* loci must have been lost [30]. The origin of this inserted region could not be determined in the initial 2015 study, as its key marker, ORF CM14, is not widespread and none of the ORF CM14-positive lineages then sequenced fit the other characteristics of the segment in question [30]. A possible explanation was that the inserted region itself was a composite or chimera consisting of many small fragments of different origins [32].

The purpose of the present study was to re-assess the evolutionary history of ST71 given the increased availability of sequencing technologies and genomic data in recent years.

## Materials and methods

### Strains and isolates

An overview of isolates and sequences considered is provided in Table 1.

**Table 1:** Isolates/sequences, their provenience and typing data

Isolate/sequence	Reference	GenBank accession	Provenience	Isolation source	Resistance genotype	MLST	MLST profile (*arc*C-L2-*aroE-glpF-gmk-pta-tpi-yqiL*)	RIDOM *spa* type	RIDOM *spa* profile	*agr* group	Capsule type
Milano-07	This study	Pending*	Mainland Italy, 2012	Bovine quarter milk sample	*mecA/C* neg.	ST71	18-1-1-1-1-5-3	t524	04–17	I	8
Milano-08	This study	Pending*	Mainland Italy, 2012	Bovine quarter milk sample	*mecA/C* neg.	ST71	18-1-1-1-1-5-3	t524	04–17	I	8
Milano-09	This study	Pending*	Mainland Italy, 2012	Bovine quarter milk sample	*mecA/C* neg.	ST71	18-1-1-1-1-5-3	t524	04–17	I	8
Milano-84	This study	Pending*	Mainland Italy, 2012	Bovine quarter milk sample	*mecA/C* neg.	ST71	18-1-1-1-1-5-3	t524	04–17	I	8
22CS0319-1	This study [34],	Pending*	Sardinia, 2021	Milk of mastitic sheep	*mecA/C* neg.	ST71	18-1-1-1-1-5-3	t524	04–17	I	8
22CS0351	This study	Pending*	Sardinia, 2022	Milk of mastitic cow	*mecA/C* neg.	ST71	18-1-1-1-1-5-3	t524	04–17	I	8
FD516C	This study	Pending*	Ireland, 2011	Bovine milk	*mecA/C* neg.	ST71	18-1-1-1-1-5-3	t524	04–17	I	8
12_306	This study	Pending*	Ireland, 2012	Bovine milk	*mecA/C* neg.	ST71	18-1-1-1-1-5-3	t524	04–17	I	8
MOK042	[31]	CP029627	Ireland, 2010	Bovine milk	*mecA/C* neg.	ST71	18-1-1-1-1-5-3	t524	04–17	I	8
MOK099	[30]	SAMN03247575, re-sequenced for this study *	Ireland, 2011	Bovine milk	*mecA/C* neg.	ST71	18-1-1-1-1-5-3	t528 or t524*	04 or 04–17**	I	8
A_SAU109	-	CP186909	Mainland Italy	Bovine milk	*mecA/C* neg.	ST71	18-1-1-1-1-5-3	t524	04–17	I	8
MOK063	[31]	CP029629	Ireland, 2010	Bovine milk	*mecA/C* neg.	ST97	3-1-1-1-1-5-3	t359	07-23-12-21-17-34-34-33-34	I	5
Wood46 (NCTC7121, NCTC10344)	[35]	LR134087, LS483324	Not recorded, earlier than 1946	Not recorded	*mecA/C* neg.	ST97	3-1-1-1-1-5-3	t359	07-23-12-21-17-34-34-33-34	I	5
NCTC9752	-	LS483310	UK, before 1955	Not recorded	*mecA/C* neg.	ST97	3-1-1-1-1-5-3	N/A	*spa* gene absent	I	5
FDA209P (ATCC6538, JCM2151)	[36]	CP123853, CP158284, AP043700	Not recorded	Not recorded	*mecA/C* neg.	ST464	3-1-1-1-29-5-3	t3297	14-12-21-17-34-34-34-33-34	I	5
NCTC4137	-	LR134091	USA, 1933	Human, compound fracture	*mecA/C* neg.	ST464	3-1-1-1-29-5-3	t934	07-23-12-34-34-34-34-33-34	I	5
IVRI-FBI-646	-	CP125746	India, 2019	Animal milk	*mecA/C* neg.	ST2219	3-1-280-168-1-5-3	t359	07-23-12-21-17-34-34-33-34	I	5
Strain-15	-	CP094925	Mexico, 2015	Milk of mastitic cow	*mecA/C* neg.	ST4552	3-1-1-37-1-5-611	t224	07-23-12-21-17-34-33-34	I	5
Milano-71	This study [37],	Pending*	Mainland Italy, 2012	Bovine quarter milk, taken for screening purposes	*mecA/C* neg.	ST3042	18-146-45-18-343-329-375	t14273	04-17-25-23-34-50-17	IV	8

*See also Supplemental file 1

**Discrepancy noted between the Illumina and Oxford Nanopore sequences, which might be a sequencing artefact, or a mutational change after many in vitro passages

Three ST71 isolates from Ireland were subjected to long-read sequencing using Oxford Nanopore Technology (ONT). This included isolate MOK099, that was previously sequenced using Illumina short-read sequencing technology (SAMN03247575, [30]), and two new isolates (FD516C and 12_306). Five isolates from mainland Italy were also ONT sequenced. Four were previously assigned by DNA microarray profiling to ST71 (Milano-07, Milano-08, Milano-09 and Milano-84) and one could not be assigned to a ST using the array data (Milano-71). As an isolate of an “unknown *agr* IV lineage” [37], it was later subjected to conventional MLST typing [18] yielding ST3042. Two ST71 isolates (22CS0319-1 and 22CS0351) were collected from a sheep and a cow, respectively, on the Italian island of Sardinia. In addition, a previously published ST71 genome sequence (Irish *S. aureus* strain MOK042, GenBank accession number CP029627.1 [31]) and three genome sequences of canonical CC97 (*S. aureus* strain MOK063, CP029629.1 [31]; strain NCTC4137, LR134091.1 and strain NCTC9752, LS483310.1) were analysed in detail.

ST71 short-read sequences from the cgMLST database at https://pubmlst.org/bigsdb?db=pubmlst_saureus_seqdef were downloaded to be used for comparison of particular aspects (see below). These are listed in Table 2.

**Table 2:** ST71 short-read sequences from the PubMLST database and their provenience

cgMLST-ID	Strain ID or Accession number	Country of origin
cgMLST-ID 50362	ASM318612v1	Ireland
cgMLST-ID 42601	Strain 682	Italy
cgMLST-ID 43035	Strain 36093	Italy
cgMLST-ID 43046	26520_14107	Italy
cgMLST-ID 42246	DS1	Austria
cgMLST-ID 42738	G07I	Switzerland
cgMLST-ID 42270	DS96	Denmark
cgMLST-ID 42368	m_MR_7M	Denmark
cgMLST-ID 14446	ERR246599	UK
cgMLST-ID 14447	ERR246600	UK
cgMLST-ID 14448	ERR246603	UK
cgMLST-ID 14480	ERR246642	UK
cgMLST-ID 15071	ERR246653	UK
cgMLST-ID 10102	ERR211696	unknown
cgMLST-ID 10148	ERR211697	unknown
cgMLST-ID 10922	ERR211682	unknown
cgMLST-ID 11916	ERR175902	unknown
cgMLST-ID 11922	ERR175921	unknown
cgMLST-ID 13145	ERR175888	unknown
cgMLST-ID 13158	ERR175924	unknown
cgMLST-ID 15002	ERR234764	unknown
cgMLST-ID 25318	ERR525080	unknown

For comparison, previously published CC97 genomes were included. Several sequences originated from identical strains, kept under different names by different laboratories. ATCC6538, FDA209P and JCM 2151 (GenBank accession numbers CP123853, CP158284, AP043700) are the same strain; and NCTC10344 (GenBank LS483324) is a variant of NCTC7121 (Wood46; GenBank LR134087). They were deliberately included to assess the variability of cgMLST markers as well as of the number of genes encoding tandem repeat proteins within a genomic island of relevance for this study (see below).

### Nanopore sequencing

All bacterial strains were cultured overnight at 37 °C on Columbia blood agar (Becton Dickinson, Heidelberg, Germany).

Genomic DNA for Nanopore MinION sequencing (Oxford Nanopore Technology, ONT, Oxford, UK) was extracted using the Nucleospin Microbial DNA Kit (Macherey Nagel, Düren, Germany). For each strain, one full inoculation loop was washed with 500 µL of 1× PBS (pH 7.4), centrifuged for 5 min at 4,000 × *g*, and resuspended in 100 µL of the kit´s elution buffer (buffer BE).

The extraction procedure followed the manufacturer’s protocol with two minor modifications: samples were lysed in a BeadBeater (BeadBug Homogenizer, Biozym, Germany) for 12 min at maximum speed, and proteinase K was subsequently inactivated by incubation at 70 °C for 5 min. After cooling, 4 µL of RNase (100 mg/mL; Sigma Aldrich, Steinheim, Germany) were added, and samples were incubated for 5 min at 37 °C. DNA was finally eluted in 70 µL of nuclease-free water (Carl Roth, Karlsruhe, Germany).

Following extraction, DNA was prepared for sequencing using the native barcoding kit 24 V14 (SQK-NBD114.24; Oxford Nanopore Technologies, Oxford, UK) in accordance with the manufacturer’s protocol. Prior to library preparation, DNA samples underwent size selection using AMPure beads (Beckman Coulter, Krefeld, Germany) at a 1:1 (v/v) ratio. Approximately 600 ng of DNA per sample, quantified with a Qubit 4 Fluorometer (Thermo Fisher Scientific, Waltham, MA, USA), were used for library preparation before loading onto an ONT flow cell (FLO-MIN114) equipped with R10.4.1 pores.

Sequencing was performed on a MinION Mk1B device for 90 h, starting with at least 900 active pores and managed through MinKNOW software (v24.11.10). Base calling was carried out using the dorado basecaller (v1.0.2) with the model es_dna_r10.4.1_e8.2_400bps_sup@v5.0.0_bacterial-methylation, generating Fastq files containing 4,000 reads each. Barcode trimming was performed with dorado demux (v0.7.3) using the same model.

Quality-filtered reads were assembled into contigs using Flye software (v2.9.5; [38]). The resulting assemblies were polished in two stages: first, with four iterative rounds of Racon (v1.5.0; [39]) using the parameters match 8, mismatch 6, gap 8, and a window length of 500; followed by a final polishing step with Medaka (v1.12.1; [40]) employing the model r1041_e82_400bps. The final corrected assemblies were subsequently used for downstream analyses.

## Results

**Fig. 1:**
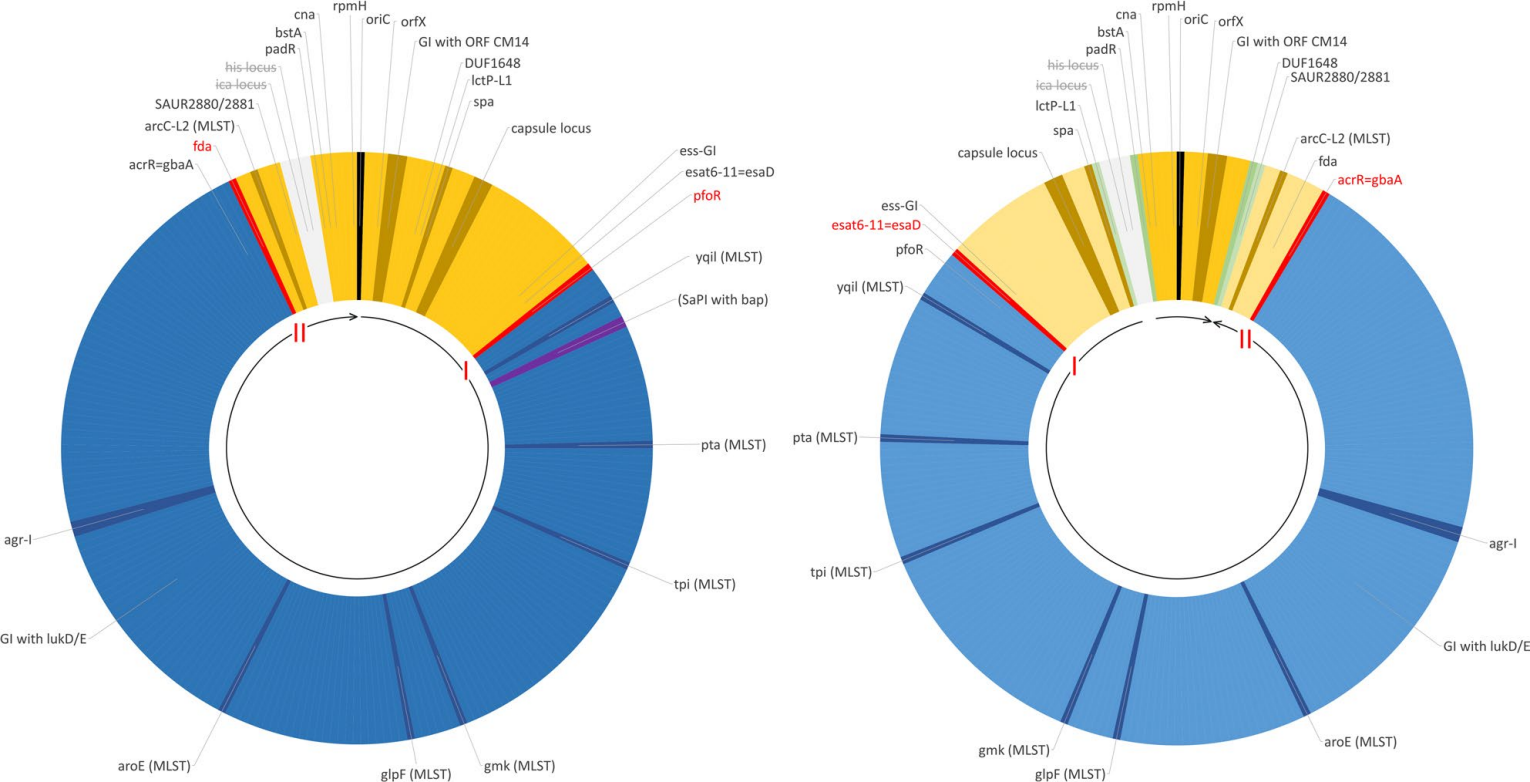
The structure of a typical ST71 (left) and a Sardinian ST71 (right) genome. Regions of CC97 provenience are shown in blue and those of ST3042 origin are shown in yellow. Inverted regions are shown in a lighter shade and are indicated by the arrows in the inner circle. Typing markers (see Table 1) are shown in darker shade. Recombination breakpoints are indicated in red and with Roman numerals; inversion breakpoints in green. The normal position of the (absent) *his* and *ica* loci is shown in grey. The position of the SaPI with *bap* (present only in two Irish isolates) is indicated in purple

Table 1 summarises the typing data for the nineteen isolates/sequences considered in detail. All ST71 isolates/sequences differed from canonical CC97 in their *arcC* allele (18 versus 3) and in the *spa* type, while ST3042 displayed the same *arcC* allele as ST71 and a related *spa* type. ST71 has the same capsule type as ST3042 but the *agr* alleles of canonical CC97. An abridged version of the cgMLST data and of data for some genomic islands are provided in Table 3. For a complete version, see Supplemental file 2. Simplified diagrams of ST71 genomes are provided in Fig. 1. A detailed description of the cgMLST data, the recombination breakpoints and the genes affected are provided in the following paragraphs.

In short, analyses by cgMLST of the core genomes of CC97, ST71 and ST3042 indicate that ST71 is derived from CC97 by insertion of a large fragment of DNA (approx. 412,500 bp) around *oriC* that originated from a donor identical or closely related to ST3042. However, there were two clearly distinct variants of ST71 that could be distinguished based on the exact localisations of the recombination breakpoints. One variant was represented by the two Sardinian isolates, while all other published and study sequences represent the other variant. One recombination breakpoint was situated within the *pfoR* (SAUR0307) gene in the majority of ST71 sequences. However, in the Sardinian sequences it bisected a gene (*esat6-11=esaD=essD*) that belongs to a genomic island around SAUR0300. The other recombination breakpoint was, in the majority of sequences, situated in the *fda* (SAUR2817) gene but could be found within *acrR=gbaA* (SAUR2789) of the Sardinian isolates. This suggested that the Sardinian ST71 emerged independently from the other ST71, by another recombination event involving the same parental strains.

All ST71 isolates lacked the genes from *ywrF* (SAUR2882) to *pcp* (SAUR2913). This deletion included the *ica* (SAUR2888 to -2892) and *his* (SAUR2895 to- 2903) operons as noted previously [30]. The ST3042 sequence showed a similar loss, although even more genes in this region, including the collagen adhesin gene *cna* (which is present in ST71 but absent in canonical CC97), were replaced by transposase genes (see below).

In the two Sardinian sequences, an inversion of a large part of the genome was observed. This affected a region between SAUR0106/SAUR0107 and SAUR2880/SAUR2914, i.e., of as much as 2.6 Mbp.

### Core genomic markers

Core genomic markers in *S. aureus* (and in related species *S. argenteus*,* S. schweitzeri* and *S. roterodami*) usually follow a fixed order, in which the numbers of the cgMLST markers from SAUR0001 to SAUR2939 essentially denote their relative position in the genome. cgMLST markers from SAUR2940 upwards were apparently included later in the scheme and, hence, they are scattered across the genome.

The numerical system of cgMLST markers provides a concise overview on the identity of genomic regions and facilitates a quick comparison of canonical CC97, ST71 and ST3042 sequences (see Table 3 for an abridged version and Supplemental file 2 for the full version). The only drawback is the fact that there are no other published genome sequences for ST3042 than the one described herein. Thus, some alleles may not be represented in the MLST database; however, if novel alleles are identified, the genes will at least be marked as “present” in Table 3, and/or alignments are provided as Supplemental files 3 and 4 to facilitate a direct comparison. There are also multiple alleles known to be associated with CC97, making it easier to find or to rule out a match to ST71 sequences. For instance, *walI=yycI* (SAUR0023) can have alleles 209 or 211 in CC97, 59 in ST71, but a novel allele is present in the single ST3042 genome sequenced, with no number yet assigned. In all ST71 sequences apart from two, cgMLST alleles from *dnaA* (SAUR0001) to A5IPH6 (SAUR0306) matched alleles from ST3042 while from Q5HJ57 (SAUR0308) until close to the end of the genome they correspond to CC97 (with *pfoR* (SAUR0307) having a unique allele, see below). The two ST71 exceptions were the two Sardinian isolates in which *dnaA* (SAUR0001) to *esat6-05=essB* (SAUR0285) match ST3042 while they corresponded to CC97 from DUF0600-TIGR01741_MW0279 (SAUR0298) onwards. This placed the putative recombination breakpoint for these two isolates into a genomic island as discussed below.

**Table 3:**
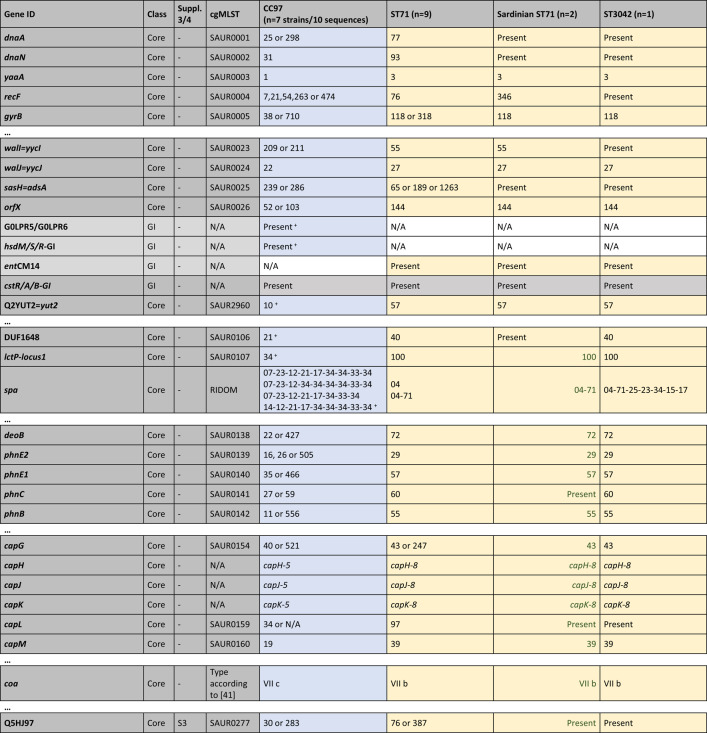

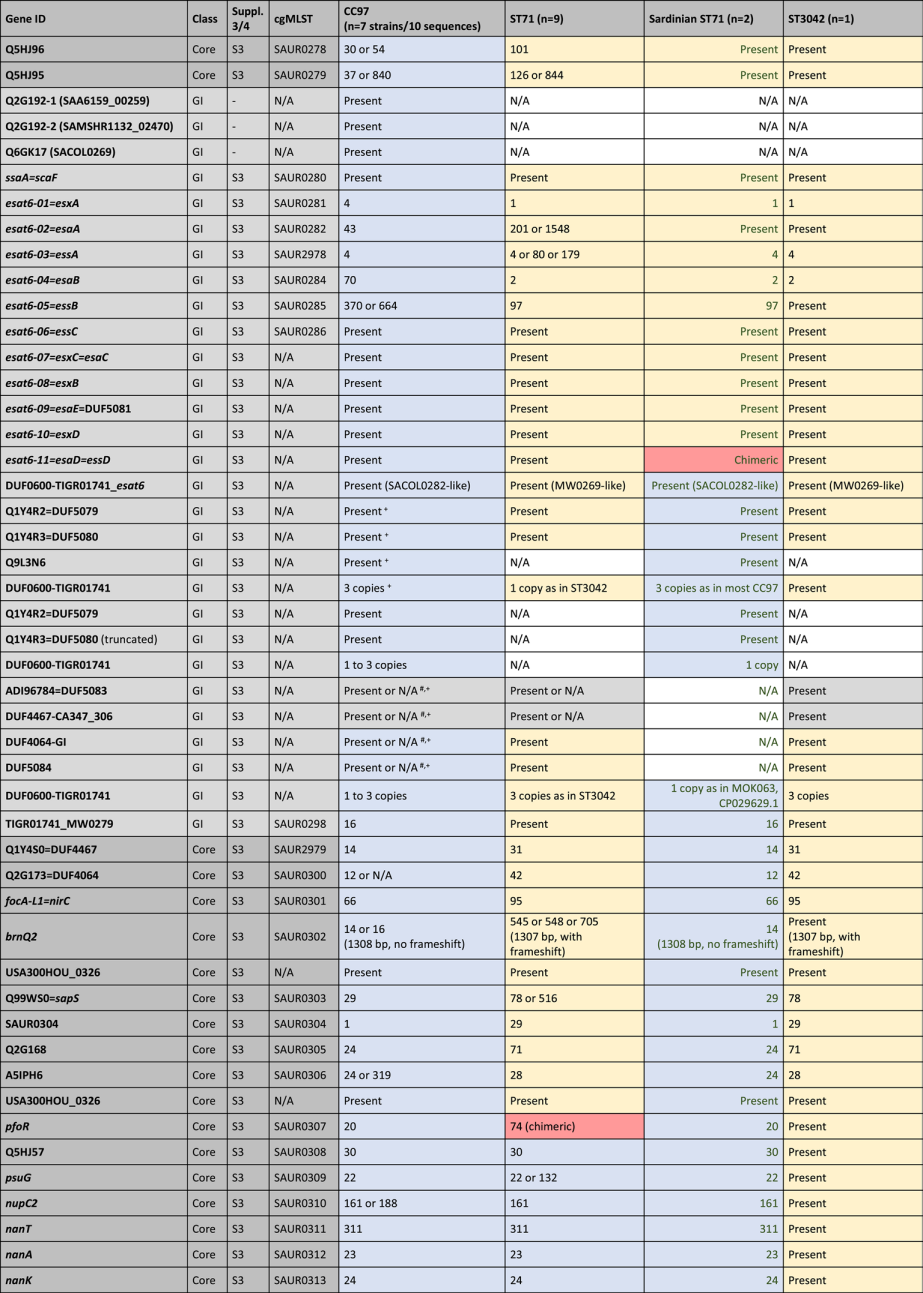

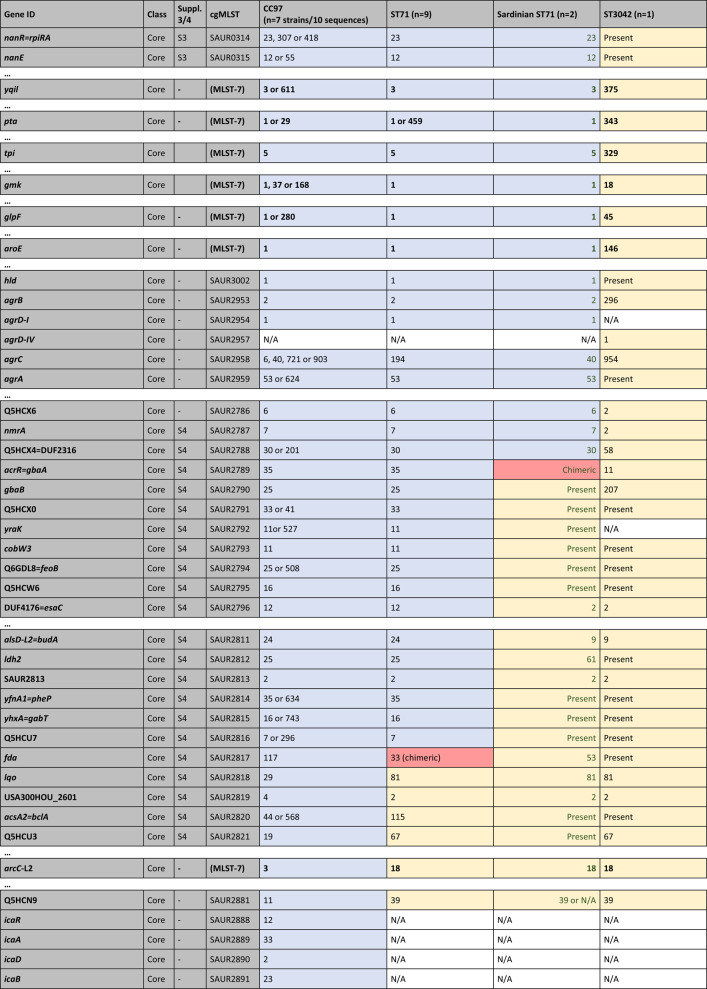

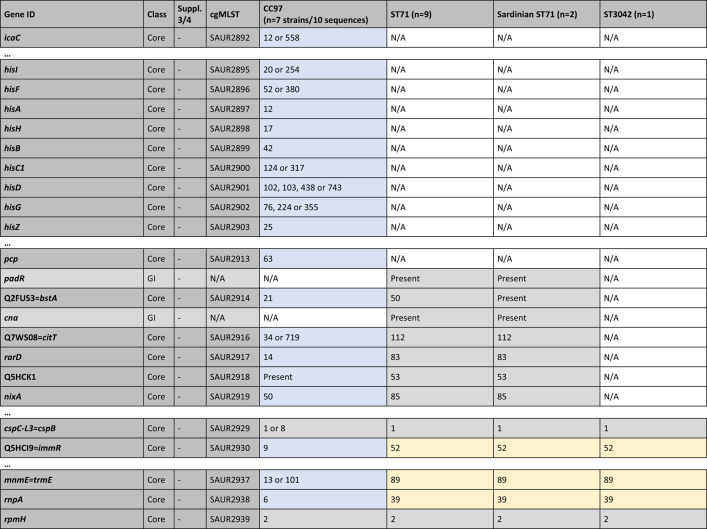
cgMLST data and some genomic islands (abridged version, for full version see Supplemental file 2). The analysis is based on the strains/sequences listed in Table 1 but the sequences from the PubMLST databases were included into underlying alignments (see Supplemental files 3 and 4). Genes are listed in the standard order, but the inverted region in Sardinian isolates is left-aligned and marked with green font. Numerical codes refer to cgMLST profiles (https://pubmlst.org/bigsdb?db=pubmlst_saureus_seqdef), except for the *spa* types (RIDOM) and the seven classical MLST markers according to Enright [18]; these are marked “MLST-7” in bold font. “Present” means cgMLST markers where the gene was detected but no code number was yet assigned. For genomic island (GI) markers, it just indicates presence. N/A means complete absence or (theoretically) truncation beyond recognition. Suppl. 3/4, S3 and S4; Sequences/alignments are provided in Supplemental files 3 and 4, respectively. Blue shade indicates CC97 provenience; yellow, ST3042; and grey, undetermined provenience (based on cgMLST or alignments in Supplemental files 3 and 4). Genes shaded red are putative chimeric genes, see the alignments in Figs. 2, 3, 4 and 5 and Supplemental files 3 and 4 [41]

^#^Absent from CC97 strain MOK063, GenBank CP029629.1

^+^Absent from CC97 strain NCTC9752, GenBank LS483310.1

For the markers downstream of SAUR0298 (Sardinian ST71 isolates) or SAUR0308 (other ST71 isolates), alleles in ST71 correspond to those in canonical CC97.

However, the “last” CC97-like gene in the two Sardinian isolates was Q5HCX4=DUF2316 (SAUR2788). The gene *acrR=gbaA* (SAUR2789) differed from both CC97 and ST3042 sequences (see below). From SAUR2790 onwards, alleles were identical or related to ST3042 as the alignment shows (see below) although there are no cgMLST allele numbers yet assigned. In all other ST71 sequences, Q5HCU7 (SAUR2816) is the last gene with an allele matching CC97. The *fda* (SAUR2817, fructose-bisphosphate aldolase class 1) allele was novel, present neither in C97 nor in ST3042. From *lqo* (SAUR2818) downstream to *oriC* alleles match ST3042.

The absence of two major gene loci from the regions between SAUR2788/2816 and *oriC* of ST71 and ST3042 will be discussed below.

### Analysis of genomic islands

*S. aureus* harbours a large number (approximately 80–100) of genomic islands (GIs), i.e., genes or clusters of genes that are frequently, but not universally present, that always inhabit the same position within the genome and whose presence is strictly linked to the clonal complex affiliation. Some GIs consist of only one gene or a small number of genes, while more than 50 different genes might be associated with some of the major GIs (although not all of them are simultaneously present). The actual composition and gene content of these major GIs depend on the clonal complex affiliation. For instance, CC5 carries the *egc* cluster and *lukD/E*, CC30 has *egc* but no *lukD/E*, CC1 and CC8 are positive for *lukD/E* and negative for *egc* while CC398 lacks both, *egc* and *lukD/E.* Thus, the presence and gene content of major GIs can be used to determine the provenience of fragments of a staphylococcal genome when comparing a fragment from a putative chimeric strain(s) to the corresponding regions of the genome of putative donors.

One GI, which is of relevance for the CC97/ST71/ST3042 issue, is located directly downstream of *orfX* (SAUR0026) and the SCC integration site (Fig. 1). It is commonly associated with the exotoxin genes *seh* and ORF CM14. Excluding genes that occur in varying positions, or in multiple copies, there are about 50 genes associated with this GI (see Supplemental file 2), whose presence or absence can be used to extract information on the provenience of the genomic region. The presence of ORF CM14 was one of the first recognised features distinguishing ST71 from canonical CC97. Indeed, ST3042 also carries this gene, as well as the 15 others that comprise this GI in ST71 (although two of them, DUF3125 and a staphylococcal interspersed repeat unit, are also present in CC97, although in other positions). Canonical CC97 lacks ORF CM14 but has another five genes (including a type I restriction-modification system) that are absent from ST71 and ST3042. Another nine genes (including the *cstR/A/B* operon) are present in CC97, ST71 and ST3042 sequences. Of note, one CC97 sequence (NCTC9752, LS483310.1; Supplemental file 2) lacks this GI and the next GI downstream as well as the *spa* gene.

**Fig. 2:**
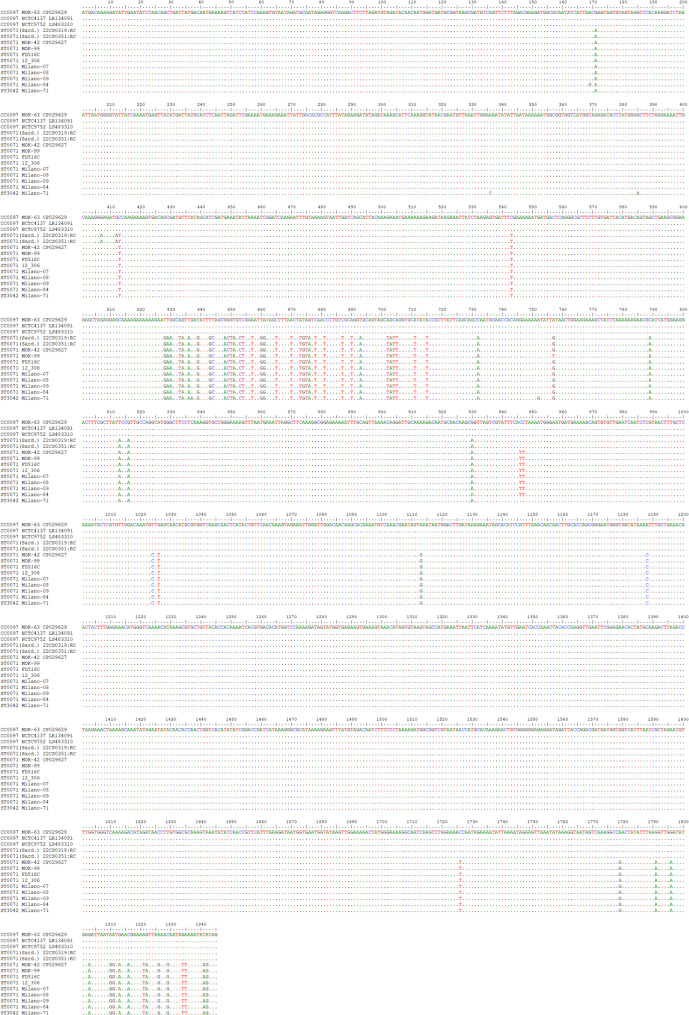
Alignment of *esat6-11=esaD=essD* from the CC97 sequences, from Sardinian ST71 (marked as “Sard.”), other ST71 and from ST3042. Alignments for this gene (and the gene shown in the following figure) that also include surrounding genes and the ST71 sequences from the cgMLST database are presented in Supplemental file 3

**Fig. 3:**
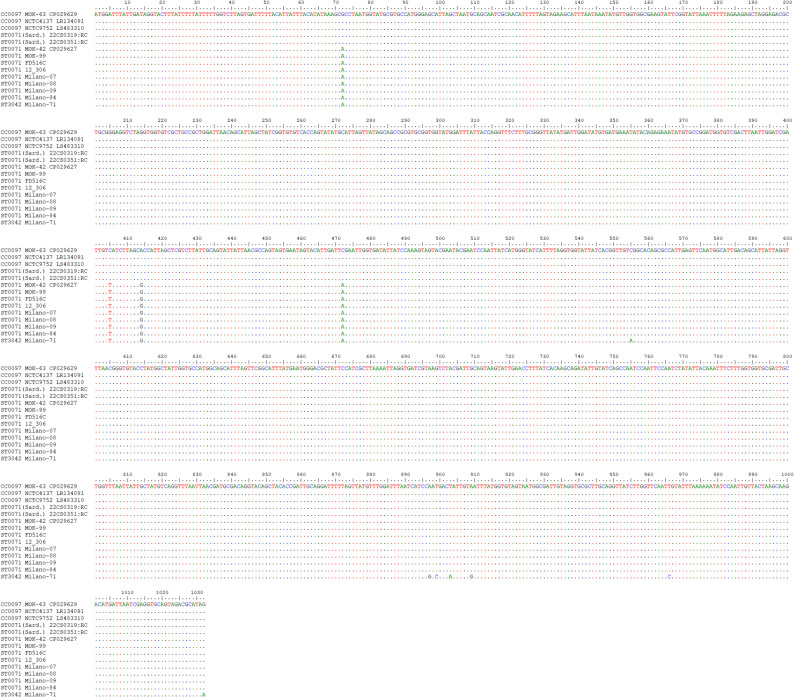
Alignment of *pfoR* (SAUR0307) from the CC97 sequences, from Sardinian ST71 (marked as “Sard.”), other ST71 and from ST3042

A second GI, directly downstream of *dusC* and accompanying genes (see Supplemental file 2), also has the same gene content in ST71 and ST3042, which differs from the corresponding GI in CC97. Some minor islands will not be discussed here in detail but are shown in Supplemental file 2. Another major GI further downstream comprises a cluster of eleven genes encoding an ESA6-like secretion system (Ess), and some of the genes were recently included into the cgMLST scheme (SAUR0280, SAUR0281, SAUR0282, SAUR2978, SAUR0284, SAUR0285, SAUR0286). Another approximately 20 genes might, or might not, be present in this GI, depending on CC affiliation. In addition, this GI includes multiple copies of a putative gene DUF0600-TIGR01741, related to MW0279, whose numbers, actual length and sequence might be variable. The Sardinian ST71 sequences matched, in this regard, one of the CC97-associated variants (MOK063) while the other ST71 sequences corresponded to ST3042 (Table 3/Supplemental file 2). An alignment of the genes (Figs. 2 and 3; Supplemental file 3) shows that one breakpoint of the large chromosomal recombination bisects one of the genes (*esat6-11=esaD=essD*) belonging to this GI, but only in the two Sardinian genomes. The alignment confirms that the exact localisation of this breakpoint in the Sardinian sequences differs from the corresponding breakpoint localisation in all other ST71 sequences (including genome sequences from the cgMLST databases). More details are discussed below.

All following GIs of ST71 either match the corresponding GIs in CC97 or are identical in all three complexes concerned. Thus, they shall not be discussed in detail here, their gene content is summarised in Supplemental file 2. Differences emerge only downstream of the second recombination breakpoint and due to large-scale deletions in ST71 and ST3042 (a detailed discussion of both issues is provided below).

### The inversion of a part of the genome in Sardinian strains

The only exceptions from the strict order of core genomic markers mentioned above are occasional inversions, in which a part of the genome was excised and re-integrated in reverse order and orientation, and hence the cgMLST code numbers are “counting backwards”. This has been described for a Russian MRSA strain (GenBank: AP017377) in which the inverted region is flanked by IS256 elements [42] and for a PVL-positive *S. argenteus*, in which the inverted region lies between two lysogenic prophage genomes [43]. Short-read (e.g., Illumina) sequencing cannot be expected always to be able to identify this phenomenon, because the natural orientation of the contigs is lost and because the flanking prophage or transposon sequences might be present in multiple copies causing contigs to end exactly at those positions that are of interest.

CC97-MSSA strain MOK063 (GenBank CP029629.1) also shows such a phenomenon, in which *lysS* (SAUR0525) is followed by SAUR2346, SAUR2345, SAUR2344 etc. until SAUR0541 after which *salA=parA* (SAUR2357) and the rest of the genome can be found in normal orientation. Here, neither prophages nor IS256 elements are noted to flank the inversion, which comprises approximately 1.67 Mbp.

The two Sardinian ST71 isolates carried such an inversion (Fig. 1). It started from SAUR0106 as the last regularly oriented and SAUR2880 (in 22CS0319-1)/SAUR2881 (in 22CS0351) as the first inverted cgMLST marker. It ended with *lctP-*1 (SAUR0107) as the last inverted gene and *padR* (situated on a GI)*/bstA*=Q2FUS3 (SAUR2914) as the first regularly orientated markers downstream, encompassing as much as 2.6 Mbp. Towards DUF1648 (SAUR0106), the inversion was in both strains flanked by four copies of an IS431 transposase plus two genes that usually are plasmid-associated. In isolate 22CS0319-1, a mobile genetic element of 14,000 bp harbouring 17 genes was additionally integrated there that removed Q5HCN9 (SAUR2881). Genes of this element were also present in other strains (ST71; Milano-84, MOK099 and CP186909 and CC97; LR134091), although in other positions. Towards *padR* and SAUR2914, the inversion was flanked by one copy of an IS431 transposase (in isolate 22CS0351) or, respectively, by three copies of an IS431 transposase plus the (normally plasmid-borne) *aurR/I/D/C/B/A/T* aureocin locus (22CS0319-1). Thus, a Sardinian ST71 genome basically can be regarded as an assembly of four distinct fragments. First, there was a ST3042-derived region with normal gene order and orientation, from *padR* and SAUR2914 through *oriC*, to SAUR0106. It was followed by another ST3042-derived fragment, in inverted order and orientation, from SAUR2881 to SAUR2790, with the “second” recombination breakpoint (see below) bisecting SAUR2789. The third fragment then consisted of genes of CC97 provenience, that again are inverted, “counting backwards” from SAUR2788 to SAUR0300 and into the *ess* GI delineated by the “first” recombination breakpoint. This was followed by another, inverted region of ST3042 descent, from GI associated *esat6-10=esxD* and SAUR0286 to *lctP-1* (SAUR0107), after which, with *padR* and *bstA*, SAUR2914 the regularly orientated ST3042 region started.

### Recombination breakpoint I

As mentioned above, one recombination breakpoint or fault line affects the major GI further comprising the *ess* gene cluster. Aligning these genes, it becomes apparent that among genes that occur in both CC97 and ST3042 genomes, specific alleles can be distinguished. This was easily visible for *ssaA=scaF*,* esat6-01=esxA*,* esat6-02=esaA*,* esat6-03=essA*,* esat6-04=esaB*,* esat6-05=essB*,* esat6-06=essC* as these genes are part of the cgMLST scheme (SAUR0280 to SAUR0282, SAUR2978, SAUR0284 to SAUR0286) yielding, when already assigned, different allele numbers (Table 3/Supplemental file 2), with ST71 isolates sharing alleles with ST3042, but differing from canonical CC97. This was also true for *esat6-07=esxC=esaC*,* esat6-08=esxB*,* esat6-09=esaE*=DUF5081 and *esat6-10=esxD*. For *esat6-11=esaD=essD*, another phenomenon could be observed (for an alignment, see Fig. 2/Supplemental file 3). Here, all ST71 sequences shared 44 single nucleotide polymorphisms (SNPs) with the ST3042 sequence, differing from canonical CC97, up to position 930 in this gene. Henceforth, Sardinian sequences were identical to CC97. All other ST71 sequences (including those from the cgMLST database) carried at positions 946/947 a double “T” which is neither present in CC97 nor in ST3042 and from a SNP in position 1024 on, they shared another 21 SNPs with ST3042, differing from the CC97 allele. For all genes downstream, the two Sardinian isolates had the same alleles as canonical CC97, while the other ST71 sequences still matched ST3042. This was also apparent by checking the cgMLST alleles for SAUR2979 and SAUR0300 to SAUR0306 (Table 3/Supplemental file 2).

CC97 and Sardinian ST71 had cgMLST allele 20 of the *pfoR* gene (SAUR0307), all other ST71 had allele 74, while for ST3042, there was no number yet assigned. SAUR0307-20 differed from SAUR0307-74 and the ST3042 allele in 4 SNPs upstream of position 850, while SAUR0307-20 and − 74 were identical henceforth, differing from ST3042 in 6 SNPs (see Fig. 3/Supplemental file 3). All following genes downstream in ST71 matched canonical CC97.

### Recombination breakpoint II

Again, the exact location of the second breakpoint in the two Sardinian isolates is different than in all other ST71 sequences, even when ignoring the issue of orientation.

**Fig. 4:**
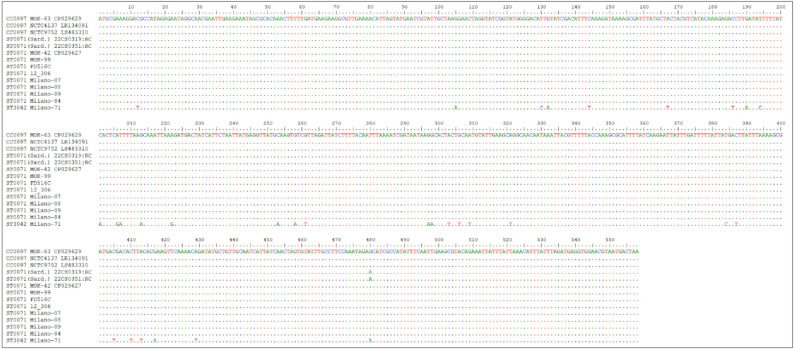
Alignment of *acrR=gbaA* (SAUR2789) from the CC97 sequences, from Sardinian ST71 (marked as “Sard.”), other ST71 and from ST3042. Alignments for this gene (and the gene shown in the following figure) that also include surrounding genes and the ST71 sequences from the cgMLST database are presented in Supplemental file 4

**Fig. 5:**
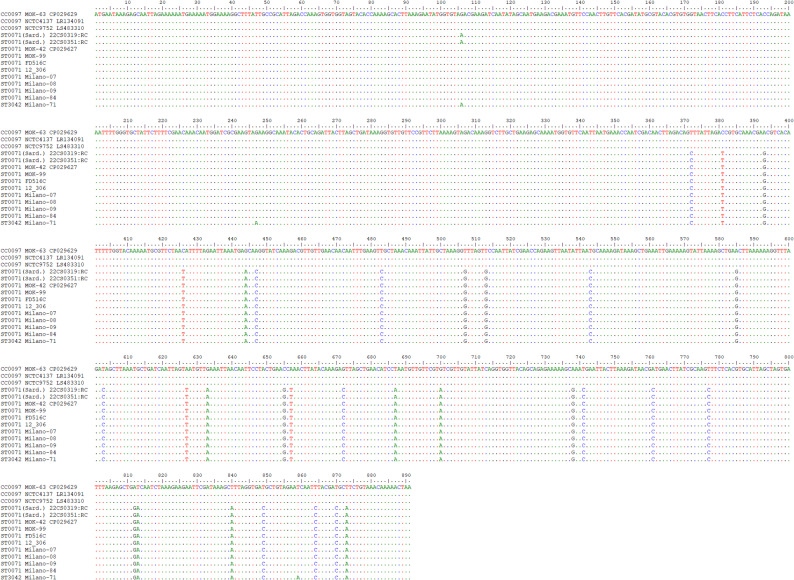
Alignment of *fda* (SAUR2817) from the CC97 sequences, from Sardinian ST71 (marked as “Sard.”), other ST71 and from ST3042

The sequences of the Sardinian isolates presented with an unassigned allele for the cgMLST marker *acrR=gbaA* (SAUR2789), encoding glucose-induced biofilm accessory regulator A. All other ST71 and CC97 carried SAUR2789-35 while ST3042 had SAUR2789-11. An alignment (see Fig. 4/Supplemental file 4) showed 30 SNPs separating all CC97 and ST71 sequences from ST3042; however, the Sardinian strains and ST3042 shared a SNP in the 3ʹ end at position 480. Differences between non-Sardinian ST71 and CC97 accumulated only downstream of *fda* (SAUR2817). CC97 had SAUR2817-117. Sardinian ST71 had SAUR2817-53 and for ST3042, no allele number was yet assigned. Non-Sardinian ST71 had allele SAUR2817-33. Upon inspection of the alignment (see Fig. 5/Supplemental file 4), this allele can be regarded as chimeric with ST71 and CC97 sequences being identical for the 5ʹ terminal positions. From position 372 to the end (at pos. 891), 30 SNPs separated ST71 from CC97, but only one from ST3042. All following genes matched, if present, ST3042 rather than CC97.

### Gene deletions in ST71 and ST3042 (*cna*, *ica* and *his* operons)

The absence of the *ica* (SAUR2888 to -2892) and *his* (SAUR2895 to -2903) operons from ST71 has been noted previously, and its histidine auxotrophy has been demonstrated [30]. Sequencing of additional isolates for the present study, including Sardinian isolates, revealed that loss of both operons is a common feature of all ST71 isolates sequenced. Indeed, they lack all genes from *ywrF* (SAUR2882) to *pcp* (SAUR2913), with the first following genes being the genomic island gene *padR* as well as *bstA*=Q2FUS3 (SAUR2914). In the Sardinian sequences, this also marked one flank of the genomic inversion described above. This region was replaced by an IS431 insertion element with a variable number of transposase genes that encoded a toxin from a plasmidic toxin/antitoxin system and one or two mobile element associated genes (corresponding to SAB1296c, SAB1297 in AJ938182.1) plus, in one case (Milano-84), an integrated plasmid. Similarly to ST71, the ST3042 isolate lacked *ica* and *his* operons. Furthermore, it also lacked the genes situated between these loci and Q6GDA0 and *cspC-L3=cspB* (SAUR2929). This included the collagen adhesin gene *cna* (which is present in ST71 but absent in canonical CC97). Here, again, three IS431 transposase gene copies, the toxin gene and SAB1296c were present. Whether this is representative for ST3042, or for a sub-lineage within ST3042, or just an accidental deletion affecting this particular isolate cannot be determined due to a lack of sequences for ST3042 in publicly available sequence databases.

### Other genes of interest

As mentioned above, one Sardinian isolate (22CS0319-1) carried the *aurR/I/D/C/B/A/T* aureocin locus [44] located on an integrated plasmid.

Two Irish isolates (FD516C_150525 and 12_306) were positive for the *bap*-gene encoding a biofilm-associated protein [45, 46] on a *S. aureus* Pathogenicity island (SaPI) located next to *guaA* (SAUR0412). The ST3042 isolate, some of the other ST71 isolates and one CC97 reference sequence (IVRI-FBI-646, GenBank CP125746) harboured another SaPI at the same position (see Supplemental File 2). This SaPI comprised, among genes for “putative proteins” and an integrase gene, also genes encoding adenosine deaminase (*add*), a veterinary paralogue of complement inhibitor SCIN family protein (*scn*2) and a van Willebrand factor binding protein (*vwb3*; corresponding to SAPIG0481 to SAPIG0483 in GenBank AM990992.1).

## Discussion

Analysis of the genomes of CC97, ST71 and ST3042 provides convincing evidence that ST71 is derived from CC97 by insertion of a large fragment of DNA (approx. 412,500 bp) originating from a donor identical or closely related to ST3042. The only major difference is the absence of *cna* and a few surrounding genes in ST3042. However, there is only one known genome sequence of ST3042, the one described herein, and it might be assumed that different strains or sub-lineages of a putative “CC3042” might differ in the extent of the deletion, with some lacking *ica* and *hi*s operons only, while others lack *ica*,* his* and *cna*. Interestingly, there are two different variants of ST71 that differ in the size and in the exact localisation of the recombination breakpoints. Given the differences between Sardinian and all other ST71 sequences (including sequences in the cgMLST database from three Italian ST71 isolates), it is reasonable to assume that ST71 emerged twice by integration of ST3042 DNA into a CC97 backbone genome.

CC97 is a globally disseminated lineage that can be found in cattle but also in small ruminants, cervids, pigs, humans and in scavenging birds (which might have been exposed to ruminant carcasses) [3, 47–58]. Essentially nothing is known about ST3042, neither its geographic distribution nor its natural history including host species. The isolate sequenced herein originated from Italian cattle. There is only one entry in the MLST database (https://pubmlst.org/bigsdb?db=pubmlst_saureus_isolates&page=info&id=5580; accessed 2025, Dec. 8th and 2026, March 10th ) and that isolate also originated from the Italian mainland. Unfortunately, there was no whole genome sequence provided, and the isolate was not available for sequencing. It is currently not known whether this lineage is restricted to Italy, and its natural host(s) remains elusive. It could be an extremely rare lineage in Mediterranean cattle. However, given the poor knowledge of methicillin-susceptible *S. aureus* in animals, it could also be a common strain in some wild animal species, possibly outside of Europe that just accidentally and sporadically got introduced into the cattle population. It might be argued that the ONT sequence described herein might be affected by sequencing errors, and that conclusions based on this sequence thus might be wrong or misleading. However, for three ST71 isolates, Illumina and ONT sequences were considered in parallel (see Supplemental file 2) and the degree of concordance was high indicating that ONT technology was sufficiently accurate. Furthermore, not only the identity of certain alleles, but also the number of genes considered as well as the mere presence or absence of genes, especially within GIs, corroborate the conclusions. However, it would be interesting to analyse more genome sequences of ST3042, or closely related STs once they become available.

It also can be speculated that most European, including Irish and British ST71 form one clade originating back to one recombination event, while a second clade resulting from another recombination might be restricted to Sardinia, where this event might have taken place. This observation warrants further work on Sardinian ruminant strains of both CC97 and ST71. Sardinian ST71 also differ from other ST71 in the orientation of a large part of the genome. As the inversion affected the ST3042-derived region, this event must have taken place later in time than the recombination of CC97 and ST3042.

The acquisitions of the aureocin locus and of the pathogenicity island carrying *bap* by some isolates emphasise the ongoing evolution of this chimeric lineage as they further contribute to fitness in the competition with other bacteria and with host defences.

A significant question is how to reconcile the ST3042 hypothesis proposed here with a concept published earlier that suggested that ST71 was a chimera consisting of a large number of small genomic DNA fragments of different origins [32]. The two proposals are not necessarily contradictory. We argue that ST3042 (or a lineage closely related to ST3042) was an *immediate* predecessor to ST71 and donor of the part of the ST71 genome that does not originate from CC97. That does not rule out that ST3042 itself might be of chimeric/mosaic nature. For instance, it might have acquired the enterotoxin homologue ORF CM14 by horizontal gene transfer from one of the few lineages that harbour ORF CM14 (e.g., CC93, CC121, CC705, CC707 and CC772) into a still-to-be-determined parent strain. The ORF CM14-carrying genomic islands of CC707 (GenBank CP029751) and CC121 (CP026067, CP022717, CP022682, CP170404.1) have, compared to ST3042, identities of 99.90% and at least 99.77%, respectively. It theoretically could even be possible that ST71 and ST3042 are both chimeric lineages that carry a fragment of the genome of the same unknown donor lineage. With an increasing availability of genomes of *S. aureus* from animal sources and more diverse geographic regions, the natural history of ST71 and ST3042 might be more clearly resolved in future.

The fact that all lineages of *S. aureus*, however genetically diverse they might be, share just four *agr* alleles and two capsule types might indicate very far-reaching intermixing by a large number of past horizontal gene transfers. Thus, we assume that more lineages than previously anticipated (see Introduction) could be regarded as chimeric comprising fragments of two or even of multiple parent strains. Of course, it becomes very complicated to analyse the provenience of regions in the genome when discussing series of several consecutive horizontal gene transfers, simply because at a certain point, distinctions between donors and recipients become blurred. In addition, the provenience of isolated genes or gene clusters consisting of only a few genes might be obscured by mutation and reversion. If two lineages shared the same cgMLST allele at one or two positions, this might be evidence of recombination, but it also could be a result of accidental mutations. If *several hundred* consecutive genes had identical cgMLST alleles, recombination is more likely than random mutation. Thus, it is easier to spot the provenience of large multi-gene segments and/or to attribute them to recombination as was indeed the case for ST71 as for any other previously described case of chimerism.

Finally, one might ask why a couple of recombination events in an obscure lineage of animal staphylococci are interesting or relevant. They are, because they shed light on the evolution of *S. aureus*, which is arguably one of the most versatile pathogens. Recombination might contribute to this success. Arguably, at least one of the recombinant ST71 clones apparently surpassed the parental lineage ST3042 in terms of abundance and geographic distribution, or at least were able to colonise European livestock, so that, from the viewpoint of a “Selfish Gene” [59], the “alliance” with genes from the pandemic CC97 lineage was a major success. The observation that ST71 emerged twice adds to the body of evidence that recombination is another, highly effective, mode of horizontal gene transfer and that it is no extraordinarily rare event in the evolutionary history of *S. aureus* but rather some kind of common process, likely to contribute to the extraordinary success of this pathogen.

## Supplementary Information

Supplementary Material 1: Supplemental file 1a: Genome sequences of study strains. Supplemental file 1b: Statistics regarding ONT sequencing of the study strains. Supplemental file 2: cgMLST data and genomic islands. Unabridged version of Table 3. Supplemental file 3: Alignment of the genes around the first recombination breakpoint. Supplemental file 4: Alignment of the genes around the second recombination breakpoint.

## Data Availability

All relevant information, including genome sequences, is present in the manuscript and in the Supplemental files. Sequences of strains have been submitted to GenBank, accession numbers pending. The BioSample accession numbers are SAMN53808998, SAMN53808999, SAMN53809000, SAMN53809001, SAMN53809002, SAMN53809003, SAMN53809004, SAMN53809005, SAMN53809006, and SAMN53809007.
