# Supplementary material for: Staphylococcus aureus sequence type 71 is a chimera that emerged twice
Source: BMC Genomics. 2026 Apr 18;27:394. doi: 10.1186/s12864-026-12777-w (PMC13093988; doi:10.1186/s12864-026-12777-w)
Supplement: Supplementary file 1 — Supplementary Material 1: Supplemental file 1a: Genome sequences of study strains. Supplemental file 1b: Statistics regarding ONT sequencing of the study strains. Supplemental file 2: cgMLST data and genomic islands. Unabridged version of Table 3. Supplemental file 3: Alignment of the genes around the first recombination breakpoint. Supplemental file 4: Alignment of the genes around the second recombination breakpoint. [file 12864_2026_12777_MOESM1_ESM.zip › Supplement 1b_Sequencing statistics.docx]

**Supplemental file 1b:** Statistics regarding ONT sequencing of the study strains.

| **Isolate ID** | **Assembly Length** | **Coverage** | **N50** | **Number of reads** |
| --- | --- | --- | --- | --- |
| **Milano-07** | 2,735,945 | 78 | 8,448 | 57,257 |
| **Milano-08** | 2,779,861 | 130 | 6,864 | 105,247 |
| **Milano-09** | 2,735,935 | 117 | 8,081 | 91,960 |
| **Milano-84** | 2,802,837 | 131 | 7,681 | 107,918 |
| **22CS0319-1** | 2,757,472 | 98 | 5,647 | 79,206 |
| **22CS0351** | 2,718,168 | 115 | 6,536 | 95,585 |
| **FD516C** | 2,237,768 | 30 | 3,521 | 36,136 |
| **12_306** | 2,853,145 | 260 | 5,656 | 289,958 |
| **MOK099** | 2,739,264 | 108 | 6,892 | 61,073 |
| **Milano-71** | 2,665,284 | 185 | 9,572 | 265,967 |
